# Detection of inversion with breakpoints in *ARSB* causing MPS VI by whole-genome sequencing: lessons learned and best practices

**DOI:** 10.3389/fgene.2024.1452498

**Published:** 2025-01-08

**Authors:** Yufeng Huang, Wenyue Deng, Hui Huang, Xiankai Zhang, Xiaohong Chen, Jian Ye, Sukun Luo, Ting Yu, Hui Yao, Hao Du, Xuelian He

**Affiliations:** ^1^ Genetics and Precision Medical Center, Wuhan Children’s Hospital (Wuhan Maternal and Child Healthcare Hospital), Tongji Medical College, Huazhong University of Science and Technology, Wuhan, China; ^2^ Department of Neurosurgery, Wuhan Children’s Hospital (Wuhan Maternal and Child Healthcare Hospital), Tongji Medical College, Huazhong University of Science and Technology, Wuhan, China; ^3^ Department of Endocrinology and Genetic Metabolism, Wuhan Children’s Hospital (Wuhan Maternal and Child Healthcare Hospital), Tongji Medical College, Huazhong University of Science and Technology, Wuhan, China

**Keywords:** mucopolysaccharidosis type VI, whole-genome sequencing, ARSB, inversion, breakpoint

## Abstract

**Introduction:**

Mucopolysaccharidosis type VI (MPSVI), an autosomal recessive lysosomal storage disorder caused by pathogenic variants in *ARSB* gene. Usually, whole exome sequencing (WES) can identify these variants, and if WES failed to detect causative variants, whole-genome sequencing (WGS) may be considered to investigate deep intronic variations and structural alterations in patients.

**Methods:**

Whole-exome sequencing (WES) and whole genome sequencing (WGS) were performed in a Chinese family having a boy with suspected diagnosis of MPS with macrocephaly, coarse facial features, broad forehead, thick lips, frontal bossing, craniosynostosis, blue spots, frequent upper respiratory infections, inguinal hernia, and dysostosis multiplex. Lysosomal enzymatic assays for leucocytes were used to assess the activity of arylsulfatase B of the boy’s leucocytes. Sanger sequencing and karyotyping analysis were used to validate the variants identified in the boy and his parents.

**Results:**

This boy diagnosed with MPSVI based on clinical phenotypes and laboratory biochemical assays, and WES identified only a maternally inherited missense variant, c.908G>T (p.Gly303Val), in the *ARSB* gene. By performing WGS, we found a paracentric inversion involving chromosome 5q14.1q13.2 (78180730-138771424 inv), disrupting the *ARSB* gene on the proband and his father. The inversion was confirmed through karyotyping analysis, and the breakpoints were validated by agarose gel electrophoresis and Sanger sequencing.

**Disscussion:**

This study reminds us that WGS should be done when WES failed to achieve a molecular diagonosis, and it also underscores the importance of WGS especially in cases of high clinical suspicion.

## Introduction

Mucopolysaccharidoses (MPSs) encompass a group of lysosomal storage disorders caused by deficiencies in lysosomal hydrolases responsible for breaking down glycosaminoglycans (GAGs) in various tissues due to defects in degradation of enzymes (Sun, 2018). Current treatment options for MPSs include hematopoietic stem cell transplantation (HSCT) and enzyme replacement therapy (ERT). In MPS type VI (Maroteaux–Lamy syndrome, MIM#253200), caused by the deficiency of arylsulfatase B, encoded by the *ARSB* gene, there are dermatan sulfate deposits (D'Avanzo et al., 2021). MPS VI is a rare progressive autosomal recessive disorder with an estimated incidence of 0.36–1.30 per 100,000 live births (Harmatz and Shediac, 2017). The diagnosis typically involves a combination of clinical features, including increased urinary excretion of dermatan sulfate, low arylsulfatase B enzyme activity in leukocytes, and identification of pathogenic variants in *ARSB* (D'Avanzo et al., 2021; Harmatz and Shediac, 2017). Clinical manifestations often include skeletal dysplasia, short stature, contractures, macrocephaly, hernias, macroglossia, hepatosplenomegaly, sleep apnea, and corneal clouding, with the onset usually in the late infantile or juvenile stage (D'Avanzo et al., 2021). To date, according to the Human Gene Mutation Database (HGMD), a total of 253 unique variants have been identified in the *ARSB* gene, predominantly consisting of missense variants (167/253 = 66%), followed by small deletions (25/253 = 9.9%), nonsense variants (12.0%), splice site and intronic variants (5.0%), small duplications (3.0%), and large deletions (3.0%). Notably, the *ARSB* gene exhibits significant genetic heterogeneity, with almost one-third (31.7%) of the unique variants reported only once and an additional 28.5% reported twice (Tomanin et al., 2018). To date, no large insertions/duplications or complex rearrangements have been reported for MPS VI.

In this study, we performed clinical and genetic evaluations of the proband, who exhibited phenotypic presentation of MPS. Through whole-exome sequencing (WES), we identified a maternally inherited variant of the *ARSB* gene in the proband. Because clinical manifestations and biochemical testing supported the diagnosis of MPS VI, further whole-genome sequencing (WGS) was performed in the proband, and a paracentric inversion of chromosome 5 was identified. This inversion disrupts the *ARSB* gene. To the best of our knowledge, this is the first report of a paracentric inversion of chromosome 5 disrupting the *ARSB* gene. The study underscores the advantage of WGS in the identification of structural variants (SVs), such as large deletions, duplications, and inversions.

## Methods

### Patients

A 1-year, 8-month-old Chinese boy was admitted owing to a suspected diagnosis of MPS. After written informed consent was obtained from the patient’s parents, peripheral blood samples were collected from the patient, as well as from his unaffected parents and older sister. The study was approved by the Ethics Committee of Wuhan Children’s Hospital (approval number 2020R006-E04).

### Whole-exome sequencing

Trio-WES and subsequent variant analysis were carried out at the medical testing laboratory (Aegicare Lab, Shenzhen, China), and the details were similar to those in a previous reported study (Sun et al., 2021). In brief, genomic DNA was isolated from peripheral blood, and the DNA was sheared into fragments of 150–300 bp using a Covaris M220 ultrasonicator (Covaris, Woburn, MA, United States). The fragmented DNA underwent end-repair and A-tailing, followed by adapter ligation and amplification. The amplified library was subjected to targeted capture using the xGen^®^ Exome Research Panel v1.0 kit (IDT, United States). Paired-end 150-bp sequencing (PE150) was performed on the Illumina HiSeq 2000 platform (Illumina, San Diego, CA, United States). Bioinformatics analysis was carried out using in-house pipeline utilizing public software and packages. The reads were first preprocessed to remove low-quality reads and adapters using fastp 0.20.1 (https://github.com/OpenGene/fastp). Then, read alignment was performed using the Burrows–Wheeler Aligner tool 0.7.17 and BWA-MEM algorithm with default parameters against the humanG1Kv37 genome reference. The generated bam files were sorted using SAMtools 1.9, and duplicates were removed using Picard 2.19.0. Germline variant discoveries were processed using the GATK HaplotypeCaller algorithm of Sentieon genomics package v.201911. Variant annotation was obtained using ANNOVAR (version 2017-07-16), which were filtered against gnomAD, Exome Aggregation Consortium (ExAC), the 1000 Genomes Project database, and NHLBI Exome Sequencing Project 6500 (ESP6500) databases. The potential impact of variants on protein functions was predicted using several software packages including PROVEAN, SIFT, PolyPhen2_HDIV, PolyPhen2_HVAR, LRT, ClinPred, CADD, MutationTaster, MutationAssessor, and FATHMM. The significance of the identified variants was classified into five categories according to the guidelines of the American College of Medical Genetics and Genomics (ACMG) and the Association for Molecular Pathology (AMP) [5]. Following the identification of a potentially pathogenic variant, Sanger sequencing was performed using custom primers to validate this variant in the patient, his parents, and his sister.

### Lysosomal enzymatic assays for leucocytes

Leukocytes were isolated with dextran from the peripheral blood of the subject. Arylsulfatase B was assessed using a colorimetric assay with dipotassium 2-hydroxy-5-nitrophenyl sulfate as the substrate.

### Whole-genome sequencing

WGS and subsequent variant analysis were carried out in the medical testing laboratory (Aegicare Lab, Shenzhen, China). Genomic DNA was extracted from peripheral blood and fragmentized using a Covaris M220 ultrasonicator (Covaris, Woburn, MA, United States), followed by library preparation. High-throughput sequencing was performed on the Illumina HiSeq 2000 platform (Illumina, San Diego, CA, United States) with 150-bp paired-end reads. Bioinformatics analysis was carried out using both public software and a self-developed pipeline. Initially, the raw data files were cleaned using fastp 0.20.1 (https://github.com/OpenGene/fastp). All cleaned data after trimming underwent alignment against the human reference genome hg19 using the BWA 0.7.17 MEM algorithm (Li and Durbin, 2009). SNPs and indels (insertion/deletion) were identified using HaplotypeCaller of GATK, followed by variant annotation through ANNOVAR (version 2017-07-16) (Wang et al., 2010), which integrated customized databases such as ClinPred (Alirezaie et al., 2018). The analysis encompassed all sequenced genomic regions, including both exonic and intronic regions with sufficient data coverage. SVs were analyzed using Delly 0.8.1 (Rausch et al., 2012), and the variants labeled as PRECISE were selected. The same process was applied to 50 samples under the same experimental conditions, and high-frequency variants were filtered based on variant co-occurrence frequency. Finally, SVs were annotated using AnnotSV 3.2.1 to identify rare inversion variants corresponding to the samples (Geoffroy et al., 2018). Variants with a minor allele frequency (MAF) greater than 1% in any of the gnomAD, ExAC, 1000 Genomes project, and ESP6500 databases were excluded from subsequent analysis. Filtering and prioritizing were then performed to look into potential detrimental variants, such as nonsense, missense, frameshift, indel, and variants impacting splice, for variant interpretation and classification according to ACMG guidelines (Richards et al., 2015).

### Sanger sequencing

To confirm the variants identified by WES or WGS, PCR was performed to amplify fragments covering the variant site in the target gene and the breakpoints of the inversion, followed by Sanger sequencing. The forward primer used for the *ARSB* gene variant c.908G > T was 5′-ACA​CAA​AAG​CTA​TCA​TTC​TTG​CTC​AAT-3′, and the reverse primer was 5′-GAC​AAA​ACA​CTC​TGA​AGG​AGC​C-3’. The forward primer used for one breakpoint was 5′-TCC​TGT​ACT​CCA​AGG​GAA​CA-3′, and the reverse primer was 5′-TGT​TTT​ACA​GGT​TTG​CCC​AT-3’. The forward primer used for the other breakpoint was 5′-ACA​CAC​AGA​TGA​GCA​AGA​TGA​C-3′, and the reverse prime was 5′-TAG​GAG​GTA​GAG​AAA​CTG​GA-3′. The PCR products were identified by agarose electrophoresis. The PCR product covering one breakpoint was 316 bp, while that of the other breakpoint was 301 bp. After Sanger sequencing, the sequences were aligned to the human reference genome sequence (GRCh37/hg19) using BLAST version 2.9.0 to confirm the breakpoints of the inversion (Camacho et al., 2009).

### G-banded chromosomal karyotyping analysis

Heparin anticoagulated blood from the patient and his parents was inoculated into the peripheral blood lymphocyte culture medium and incubated at 37°C for 68–72 h at a constant temperature, followed by continued culture with colchicine for 2 h. Lymphocytes were collected for G-banding karyotype analysis. Twenty cells in the metaphase were selected for G-banding karyotype analysis in the patient. In the case of a small proportion of chimeric karyotypes, 50–100 cells in the metaphase were selected. If the sex chromosome number was abnormal, more than 100 cells in metaphase were selected. The karyotype analysis results were interpreted based on the guidelines outlined in the “Establishment of the International System for Naming Human Cellular Genetics” (ISCN2016).

## Results

### Clinical report

**FIGURE 1:**
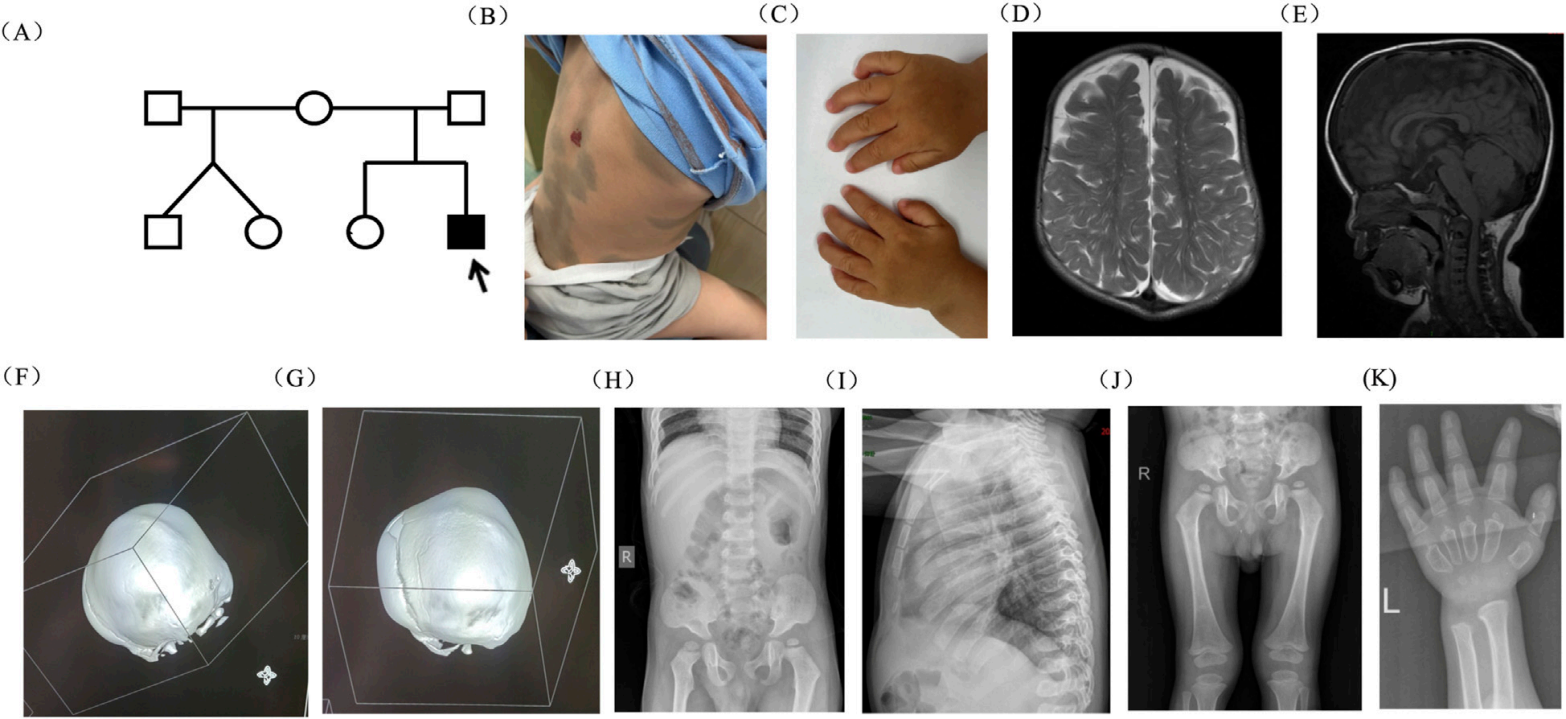
Pedigree of the family and clinical features of the proband. **(A)** Pedigree of the family. **(B)** Mongolian blue spots and hemangioma on the proband’s skin. **(C)** Claw-shaped hands. **(D, E)** MRI scan of the brain revealed abnormal head shape, enlarged perivascular spaces, enlarged bilateral frontal extra brain space, and abnormalities in the anterior longitudinal fissure. **(F, G)** The 3D bone window showed that the sagittal suture and herringbone suture were closed, while the occipitomastoid suture and temporal squamous suture were partially closed. **(H–K)** X-ray examination revealed that vertebral morphology was irregular, ribs were widened, and the posterior ribs were gradually widened like oars.

The proband, a 1-year 8-month-old boy, presented with an abnormal head shape since the age of 1 year. He was the second child of healthy, non-consanguineous parents with no history of inherited diseases. Born at 40 weeks of gestation, he experienced oligohydramnios and maternal diabetes during pregnancy. Twins born from the proband’s mother’s previous marriage to another man were reported as normal (Figure 1A). As a newborn, he was hospitalized due to hypoglycemia, cyanosis, and hyperbilirubinemia. At 12 days old, he was readmitted due to pneumonia, jaundice, and congenital heart disease. At 2 months old, he displayed internal rotation of the right foot, normal muscle tone in his limbs, absent pathological reflexes, and a tense, bulging anterior fontanelle. Heart ultrasound reexamination revealed a ventricular septal defect and moderate mitral regurgitation. The proband experienced multiple hospitalizations for pneumonia. Motor development was slightly delayed; he could sit independently at 10 months and walk independently at 1 year and 4 months of age. At 8 months, he exhibited an abnormal head shape, characterized by a prominent forehead protrusion, which became more pronounced over time. Laparoscopic inguinal hernia repair surgery was performed at 1 year and 3 months of age. At 1 year and 8 months of age, he was admitted to the Neurosurgery Department due to the worsening abnormal head shape. During hospitalization, physical examination revealed short stature, low nasal bridge, thick lips, macrocephaly, elongated anterior–posterior diameter, open anterior fontanelle, yellow hair, thick eyebrows, and stiff finger joints. Mongolian blue spots were observed on the trunk and back, along with a 1*2-cm hemangioma on the back (Figure 1B). His hands exhibited claw-like deformities (Figure 1C). Magnetic resonance imaging (MRI) revealed an abnormal head shape with a slight widening of bilateral frontal areas; abnormal signal shadows in the center of the bilateral semiovale and around the lateral ventricles, indicative of enlarged perivascular spaces; as well as bilateral frontal extra brain space and abnormalities in the anterior longitudinal fissure (Figures 1D, E). Computed tomography (CT) revealed the premature closure of cranial sutures, slightly widened frontal and temporal sulci bilaterally, and downward displacement of the lower edge of the cerebellar tonsils (Figures 1F, G). X-ray examination revealed irregular vertebral morphology and widened ribs, with posterior ribs resembling oars (Figures 1H–K). Based on the clinical presentation, MPS was suspected.

### Point variant in *ARSB* of the family

**FIGURE 2:**
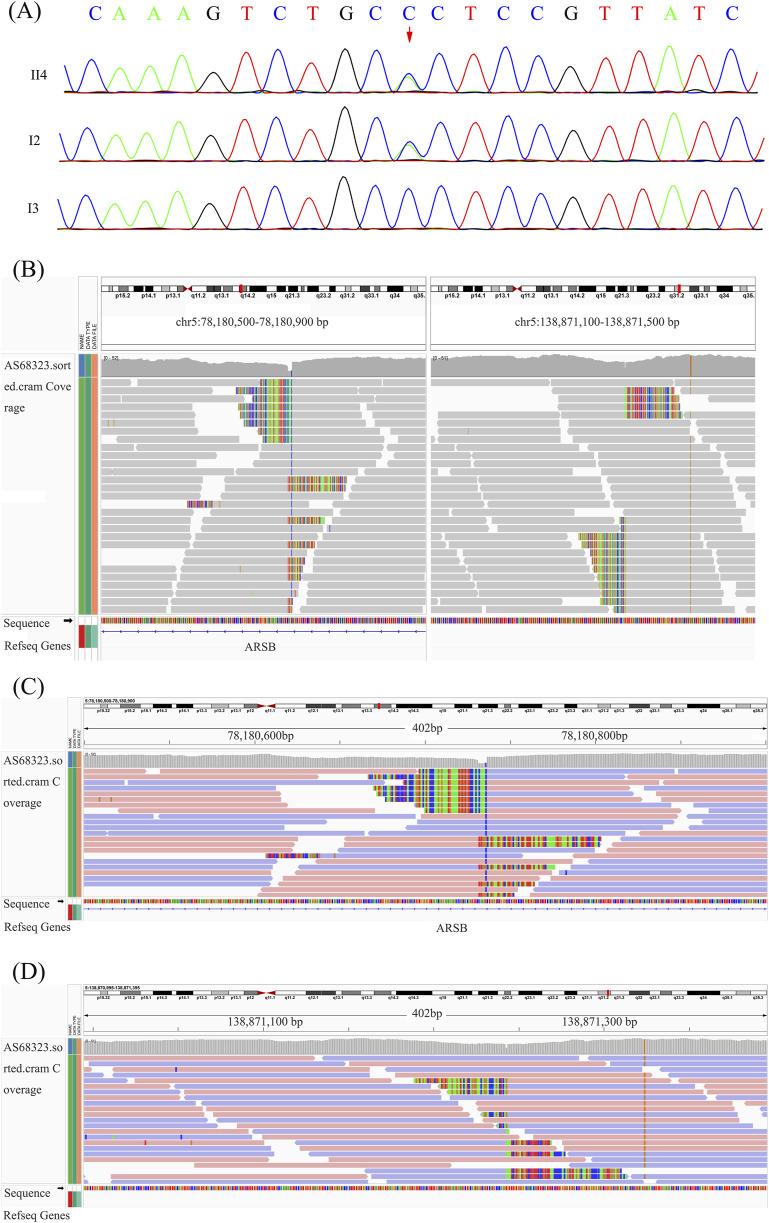
Point variant validation, breakpoints suggested by WGS, and breakpoint validation. **(A)** Sanger sequencing of the *ARSB* p.Gly303Val variant in the proband. **(B)** Overview of the two breakpoints after analyzing the original BAM file of the proband. **(C, D)** Breakpoint analysis based on the WGS data.

By using Trio-WES, a heterozygous missense variant, c.908G > T (p.Gly303Val), located in exon 5 of the *ARSB* gene (NM_000046.5), was identified in the proband and his mother but not in his father, which was validated by Sanger sequencing (Figure 2A). According to ACMG guidelines, this variant was classified as likely pathogenic variant, fulfilling criteria PM1 (occurs within a mutational hotspot), PM2-supporting (rarity in population databases), PM5 (the missense variant p.Gly303Glu classified as a pathogenic variant), and PP3 (*in silico* pathogenicity predictions).

### Deficiency of arylsulfatase B in the blood of the proband

Based on the genetic testing and clinical features, we assessed the activity of arylsulfatase B of the proband’s leukocytes and found that it was 0 nmol/h/mg Pro, which was below the detection limit of our experimental assay (normal reference range: 70.1–338.0 nmol/h/mg Pro).

### Paracentric inversion of chromosome 5 interrupts the *ARSB* gene of the proband and the father

Clinical characteristics and biochemical testing indicated the possibility of MPS VI, and only one variant of *ARSB* was identified by WES. Therefore, further WGS was performed to investigate genomic point variants and SVs or copy number variants (CNVs), such as large deletions, duplications, and inversions. After analyzing WGS data, a paracentric inversion of chromosome 5 disrupting the *ARSB* gene was found in both the proband and his father (inv5q14.1q13.2, 78180730-138771424 inv) (Figures 2B–D).

### Additional confirmation of the inversion and breakpoints

**FIGURE 3:**
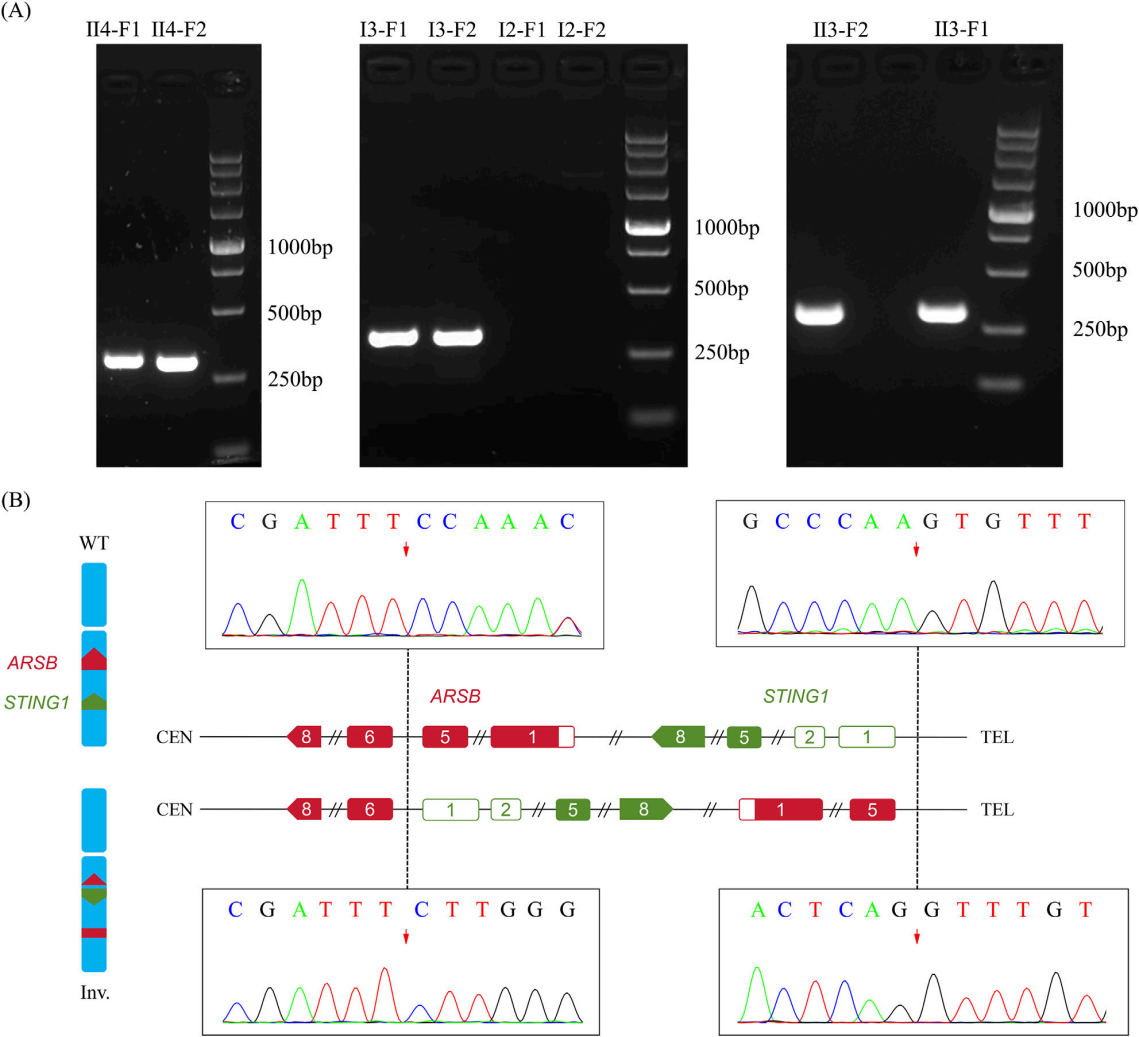
Breakpoint validation by agarose gel electrophoresis. **(A)** Agarose gel electrophoresis of PCR products of the two breakpoints. F1, left breakpoint; F2, right breakpoint; M, marker. **(B)** Diagram of breakpoints and validation of the breakpoints by Sanger sequencing.

**FIGURE 4:**
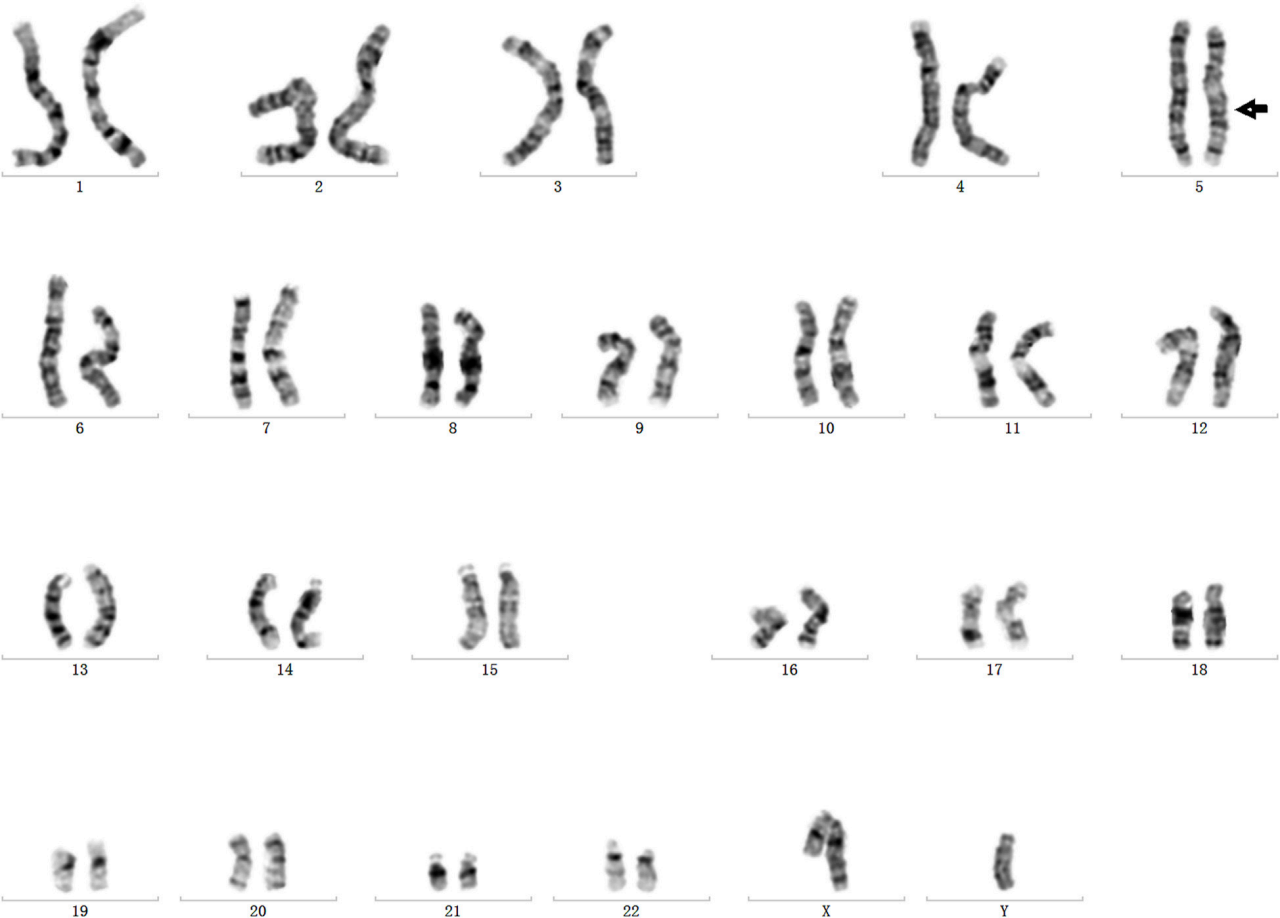
Inversion validation by chromosome karyotype analysis.

In order to validate the paracentric inversion and define the breakpoint of the inversion, agarose gel electrophoresis and Sanger sequencing were conducted. These findings indicated one breakpoint at chr5: 78180730 and chr5: 138871242 and the other one at chr5: 78180737 and chr5: 138871244 (Figures 3A, B). The inversion was further confirmed by karyotype analysis, revealing a karyotype of 46, XY, inv(5) (q14.1q13.2) (Figure 4).

## Discussion

In this study, we report a proband with a suspected diagnosis of MPS, who was found to carry a maternally inherited missense variant in *ARSB* identified by WES and a paternally inherited paracentric inversion of chromosome 5 disrupting the *ARSB* gene by further WGS. This is the first study to report a paracentric inversion of chromosome 5 disrupting the *ARSB* gene in a patient with MPS VI.

The diagnosis of MPS VI relies on enzymatic assays and genetic testing. Our patient, a 1-year, 8-month-old boy, displayed typical clinical features of MPS. Initially, Trio-WES was performed, and only one pathogenic variant was identified, and further WGS also failed to find the other variant. However, arylsulfatase B activity assessment in the proband’s leukocytes revealed that the activity was below the detection limit. In order to identify the other pathogenic variant, WGS data were reanalyzed, and the structural inversion was detected. These findings, coupled with distinctive physical features, prompted the suspicion of MPS VI, highlighting the importance of comprehensive diagnostic approaches and bioinformatics analysis in delineating disease severity and phenotype.

To date, over 200 pathogenic variants have been identified, predominantly missense variants (D'Avanzo et al., 2021); however, structural alterations in *ARSB* have not been documented. Here, we present a case of MPS VI in a patient with compound heterozygous pathogenic variants in *ARSB*: a missense variant (c.908G > T, p.Gly303Val) and a paracentric inversion disrupting intron 5 of *ARSB*. Chromosome rearrangements, such as inversions, can occur with or without causing phenotypic abnormalities and are often associated with gene damage or disturbances in genome regulation. Identifying these rearrangements accurately is challenging but crucial for understanding their impact (Hysert et al., 2006; Pons et al., 2019; Wang et al., 2023). Paracentric inversions, in particular, are common and may be underestimated in prevalence. Advanced sequencing techniques like next-generation sequencing (NGS) offer promising avenues for detailed analysis, although challenges remain in interpreting results. Despite their rarity, paracentric inversions can have significant clinical implications, emphasizing the need for thorough diagnostic approaches such as WGS (Pons et al., 2019). Additionally, while WES is adept at revealing point variants, small insertions, and deletions in exonic regions, it may not accurately detect SVs and CNVs. In contrast, WGS can effectively detect a wider range of variations, including variants in intron, SVs, and CNVs, making it a valuable tool in comprehensive genetic analysis (Kariv et al., 2022). In this study, WES initially identified a missense variant c.908G > T (p.Gly303Val) in exon 5 of the *ARSB* gene (NM_000046.5) inherited from his mother. Subsequent WGS revealed an inversion variant (chr5:78180730-138871242) interrupting the *ARSB* gene, confirmed by karyotype analysis and Sanger sequencing, inherited from the father. Obtaining a molecular diagnosis for MPS VI, as well as any single-gene defect, can be highly advantageous in facilitating familial genetic counseling.

In summary, this study emphasizes the combined application of multiple assays, including biomedical and genetic tests, and also underscores the advantage of WGS in detecting the SVs, such as large inversion. Once a molecular diagnosis is established, appropriate genetic counseling options, such as pre-implantation genetic diagnosis or prenatal testing, can be offered to families. In addition, this case emphasizes the significance of integrating clinical information, biochemical assays, and genetic information to achieve accurate diagnoses and provide optimal patient care.

## Patient consent statement

Written informed consent was obtained from the patients’ parents.

## Permission to reproduce material from other sources

No reproduced material needed copyright is involved.

## Data Availability

The datasets presented in this study can be found in the Clinvar database, accession numbers VCV003391172.1 and SCV005437156.
